# Real-world outcomes of intravitreal bevacizumab treat-and-extend for cystoid macular oedema secondary to central retinal vein occlusion

**DOI:** 10.1007/s10792-023-02811-1

**Published:** 2023-07-22

**Authors:** David Gildea, Bobby Tang, Caroline Baily, Andrea Ryan

**Affiliations:** Royal Victoria Eye and Ear Hospital, Adelaide Road, Dublin 2, D02 XK51 Ireland

**Keywords:** Central retinal vein occlusion, Cystoid macular oedema, Anti-vascular endothelial growth factor, Treat and extend, Real world

## Abstract

**Introduction:**

The purpose of this study was to report the real-world treatment outcomes using a treat-and-extend intravitreal bevacizumab protocol in cystoid macular oedema (CMO) secondary to central retinal vein occlusion (CRVO).

**Methods:**

We conducted a retrospective case series of consecutive adult patients with CMO secondary to CRVO who presented between 1st January 2019 and 31st December 2021. All included patients were treated with bevacizumab using a treat-and-extend protocol, were followed up for a minimum of 6 months and had a clinical examination including best-corrected visual acuity (BCVA) and optical coherence tomography (OCT) at every visit. The primary outcome measure was mean change in BCVA.

**Results:**

Thirty-three eyes of 33 patients were included in the study. The mean change in BCVA from baseline was + 24.5 (Median 18, SD 21.5) letters, with a mean follow-up duration of 18.5 (SD 8.9) months. The mean number of injections was 9.5 (SD 1.9) in year 1 and 7.8 (SD 2.8) in year 2. 87.9% of patients were still requiring active treatment, with a maximum interval achieved of 4-weekly in 18.2%, 6-weekly in 42.4%, 8-weekly in 6.1%, 10-weekly in 15.2%, and 12-weekly in 6.1%. The mean maximum interval achieved of those requiring ongoing treatment was 6.8 (SD 2.4) weeks. Multiple regression analyses showed that a higher baseline BCVA was negatively associated with mean visual acuity gain (*P* < 0.001) and positively associated with final BCVA (*P* < 0.001).

**Conclusion:**

The use of intravitreal bevacizumab in a treat-and-extend regimen is effective in treating CMO secondary to CRVO, in a real-world setting.

## Introduction

Central retinal vein occlusion (CRVO) is a common retinal vascular disease, with a prevalence of 0.08% in persons aged over 30 years. [1] 1] Cystoid macular oedema (CMO) is a common complication of this condition and is the primary cause of reduced vision [1].

In the treatment of CMO secondary to CRVO, intravitreal anti-vascular endothelial growth factor (anti-VEGF) agents: Ranibizumab, aflibercept, and unlicensed bevacizumab have consistently been shown to be efficacious in improving both anatomical and functional outcomes for patients [3–7].

A number of studies have used a *pro re nata* (PRN) dosing regimen; which requires a loading phase of 4-weekly injections, followed by 4-weekly patient assessments including best-correct visual acuity (BCVA) and optical coherence tomography (OCT) before a decision is made to treat at that visit or observe until the next review [3–7].

“Treat-and-extend” is an alternative treatment protocol whereby patients receive injections at every visit, using an individualised regime. After a loading phase of 4-weekly injections, treatment intervals are extended incrementally if functional (BCVA) and anatomical (OCT) parameters are stable. If parameters deteriorate, treatment intervals are reduced. This regime has demonstrated good treatment outcomes, with a lessened visit and treatment burden, making it a viable option in real-world clinical practice [8, 9].

Previous real-world studies using ranibizumab and aflibercept PRN dosing regimens have demonstrated clinically meaningful visual acuity gains, although more modest than those observed in clinical trials [10–12]; and real-world evidence for aflibercept treat-and-extend has also been published with outcomes approaching those achieved in clinical trials [13].

The aim of our study was to assess the functional outcomes of patients with CMO secondary to CRVO at a tertiary care centre in Ireland. To our knowledge, this is the first study to evaluate real-world treatment outcomes using a treat-and-extend intravitreal bevacizumab protocol in CMO secondary to CRVO.

### Methods

This was a retrospective case series of consecutive adult patients with CMO secondary to CRVO treated with Bevacizumab, using a treat-and-extend protocol, who presented between 1st January 2019 and 31st December 2021. All cases were managed in the Macular Unit in the Royal Victoria Eye and Ear Hospital (RVEEH) in Dublin, Ireland.

The following inclusion criteria were applied; (i) newly-diagnosed and treatment naïve CMO secondary to CRVO, (ii) treated with bevacizumab only, using a treat-and-extend protocol, (iii) a minimum of 6 months treatment. No patients were treated with intravitreal steroid or macular laser in this study. Of note, we did not exclude patients on the basis of baseline BCVA or degree of retinal non-perfusion.

Ultra-widefield fundus fluorescein angiography (UW-FFA) was performed on every patient within 3 months of diagnosis. We used an FFA-based definition of perfusion status; defining a CRVO as ischaemic if there were more than 75-disc areas of non-perfusion on the total widefield image, or more than 10-disc areas of non-perfusion at the posterior pole on investigators assessment [14]. Pan-retinal photocoagulation (PRP) was only performed if a patient developed neovascular complications.

Patients received a loading phase of three 4-weekly injections of intravitreal bevacizumab 1.25 mg. At every visit BCVA was measured using the Early Treatment Diabetic Retinopathy (ETDRS) chart, and OCT obtained using Spectralis™ Spectral Domain-Optical Coherence Tomography (SD-OCT, Heidelberg Engineering GmbH, Heidelberg, Germany). In addition, patients with ischaemic CRVO had an undilated slit-lamp examination at each visit including gonioscopy to assess for rubeosis. At the fourth visit (week 16) the patient was assessed as follows.

If the BCVA had improved to a maximum and the OCT was dry, the patient received an injection and was subsequently treated using a treat-and-extend regime by extending the interval in 2-weekly increments, up to a maximum of 12 weeks. If intraretinal or subretinal fluid recurred between injections, the interval was reduced back to the previous interval at which the OCT was dry.

If mild extrafoveal intraretinal or subretinal fluid persisted, but stability at maximum BCVA was maintained for three consecutive visits, the treatment interval could be extended in 2-weekly increments also. The interval was reduced back to the previous interval at which stability had been maintained if intraretinal or subretinal fluid increased (> 50micron retinal thickening effect) or the BCVA reduced by more than 5 letters.

As such, patients were identified as being treated to “dryness” or “stability”.

If the OCT remained dry or stability was achieved at a 12-weekly interval, treatment cessation with continued monitoring on a PRN basis could be considered. If a patient missed or could not attend an appointment, a further visit was arranged within 1 to 2 weeks.

The primary outcome measure was the mean change in BCVA (ETDRS letter score) from baseline to the last visit. Secondary outcome measures included the number of visits/injections required in years 1 and 2, the proportion of patients successfully completing treatment, the maximum interval achieved of those still requiring treatment, proportion of patients developing neovascular complications, and differences in outcomes depending on perfusion status.

Regression analyses were performed to assess the predictors of BCVA gain, and BCVA at the last visit. The parameters assessed were; age, gender, baseline BCVA, follow-up duration, dryness on OCT, development of neovascular complications, time from diagnosis to commencement of treatment, number of injections in year 1, and perfusion status. All variables which were significant with P < 0.05 in simple regression analysis were included in a multiple regression analysis.

## Results

Baseline characteristics of patients are presented in Table 1. Overall, 33 eyes of 33 patients were included in the study. The mean age at diagnosis was 66.2 (SD 12.5) years. 63.6% of patients were male and 30.3% of cases were defined as ischaemic. The mean BCVA at baseline was 42.3 (Median 53, SD 28.3) letters and the mean duration of follow-up was 18.5 (SD 8.9) months. The mean time from diagnosis to commencement of treatment was 4.3 (SD 2.4) weeks.

**Table 1:** Baseline characteristics

Baseline characteristic	All (*n* = 33)
Age, mean (SD)	66.2 (12.5)
*Gender* Male (%) Female (%)	63.6 36.4
Baseline BCVA, mean (SD)	42.3 (28.3)
Follow-up duration (months), mean (SD)	18.5 (8.9)
*Ischaemic Status* Ischaemic (%) Non-ischaemic (%)	30.3 69.7
Time from diagnosis to treatment (weeks), mean (SD)	4.3 (2.4)

### Primary outcome

The mean BCVA at the last visit was 66.8 (Median 75, SD 19.3) letters, representing a gain in visual acuity of 24.5 (Median 18, SD 21.5) letters. Overall, 20 patients (60.6%) achieved a ≥ 15 letter gain and only 1 patient lost ≥ 5 letters.

### Secondary outcomes

26 patients completed at least 1 year of follow-up, and 15 patients completed at least 2 years. Amongst these patients, the mean number of visits/injections was 9.5 (SD 1.9) in year 1 and 7.8 (SD 2.8) in year 2.

12.1% of patients successfully completed treatment, having remained dry on OCT or achieved stability at a 12-weekly interval. 87.9% of patients were still requiring active treatment—with a maximum interval achieved of 4-weekly in 18.2%, 6-weekly in 42.4%, 8-weekly in 6.1%, 10-weekly in 15.2%, and 12-weekly in 6.1%. The mean maximum interval achieved of those requiring ongoing treatment was 6.8 (SD 2.4) weeks. 45.5% of patients were treated to anatomical “dryness” on OCT, while 54.5% were treated to “stability”.

Of the 10 patients defined as ischaemic using our FFA-based definition of perfusion status, neovascular complications occurred in 2 (20%). Both developed anterior segment neovascularization only—one at initial presentation, and the other 18 weeks after completing treatment. Both were treated promptly with full PRP. 7 patients (70%) were still receiving active treatment. 1 patient had successfully completed treatment, and had not developed neovascular complications at 14 weeks follow-up.

Non-ischaemic CRVOs had a baseline mean BCVA of 53.9 (SD 23.5) letters and a final BCVA of 72.7 (SD 17.4) letters, representing a gain in visual acuity of 18.8 (± 20.5) letters. Ischaemic CRVOs had a baseline mean BCVA of 15.8 (SD 17.8) letters and a final BCVA of 53.2 (SD 17.1) letters, representing a gain in BCVA of 37.4 (SD 18.6) letters.

The results of the regression analyses are shown in Tables 2 and 3. Higher baseline BCVA was a negative predictive factor for mean visual acuity gain (*P* < 0.001), likely due to a ‘ceiling effect’, and a positive predictive factor for final BCVA (*P* < 0.001). Ischaemic CRVOs were more likely to improve their mean BCVA and more likely to have a worse final BCVA, although this was not significant in the multiple regression analyses due to its confounding effect with baseline BCVA. Other parameters assessed as potential predictors were not significant in our analyses.

**Table 2:** Effect of potential predictors of BCVA gain

Parameter	Simple regression		Multiple regression	
	Regression coefficient	*P* Value	Regression coefficient	*P* Value
Age	− 0.244	0.432		
Female	7.80	0.323		
Baseline BCVA	− 0.56	< 0.001	− 0.599	< 0.001
Follow-up duration	− 0.12	0.785		
Treated to Dryness	4.79	0.532		
Neovascular Complications Present	14.93	0.348		
Weeks from Diagnosis to Treatment	− 0.62	0.707		
No. of Injections in Year 1	0.309	0.734		
Ischaemic status	18.57	0.02	− 4.248	0.566

**Table 3:** Effect of potential predictors of final BCVA

Parameter	Simple regression		Multiple regression	
	Regression coefficient	*P* Value	Regression coefficient	*P* Value
Age	− 0.441	0.136		
Female	− 5.43	0.446		
Baseline BCVA	0.445	< 0.001	0.445	< 0.001
Follow-up duration	0.336	0.388		
Treated to dryness	11.633	0.085		
Neovascular complications present	− 13.08	0.361		
Weeks from diagnosis to treatment	− 0.798	0.587		
No. of Injections in Year 1	0.143	0.862		
Ischaemic status	− 19.50	0.02	− 4.248	0.566

No cases of endophthalmitis, uveitis or cardiovascular events were reported during our study period.

## Discussion

This study supports the effectiveness of a treat-and-extend regimen using intravitreal bevacizumab in patients with CMO secondary to CRVO. Our patient population achieved a mean BCVA gain of 24.5 letters which is higher than reported in other real-world studies [10–12, 15, 16].) This may be explained by the relatively high number injections administered in our study, with a mean of 9.5 (SD 1.9) visits/injections in year 1 and 7.8 (SD 2.8) in year 2. The previously published real-world study of aflibercept treat-and-extend in CMO secondary to CRVO demonstrated a mean BCVA gain of 15.1 (SD 20.2) letters at 52 weeks, from a baseline mean BCVA of 44.7 (SD 20.0) letters, achieved with a mean of 8.0 (SD 2.6) injections [13]. In another large retrospective study of 5300 patients with CMO secondary to CRVO and treated with intravitreal anti-VEGF therapies, BCVA gain at 1 year was 7.1 letters (95% CI 6.31 to 7.95) from a baseline BCVA of 39.5 letters [16]. The LUMINOUS real-world study of ranibizumab demonstrated a mean BCVA gain of 10.8 (SD 19.7) letters at 1 year, from a baseline mean BCVA of 40.6 (SD 23.9) letters, achieved with a mean of 5.4 (SD 2.7) injections [10].

In addition, the distribution of our BCVA data was negatively skewed. 30.3% of cases were ischaemic, among whom the mean baseline BCVA was 15.8 letters. As such, our reported median BCVA gain of 18 letters may be more appropriate.

87.9% of patients in our study were still requiring ongoing intravitreal injections at the end of the study period. This is similar to a real-world study of anti-VEGF agents in which 35% of eyes had persistent macular oedema after 5 years of treatment and only 14% discontinued anti-VEGF therapy within 2 years [15]. Thus, a treat-and-extend regimen is the preferred option for the majority. This is further reinforced by previous clinical trial evidence showing a reduction in BCVA gains when switching from a fixed dosing regimen to a PRN regimen [17, 18].

Of those still requiring treatment, the mean treatment interval was 6.8 (SD 2.4) weeks. Overall, 18.2% of patients remained on a 4-weekly interval, with just 6.1% achieving a 12-weekly interval. This is comparable with the CENTERA study of a treat-and-extend regimen using intravitreal aflibercept, where the mean treatment interval was 7.6 (SD 1.9) weeks and 36.9% achieved a planned interval of ≥ 12 weeks [8]. This suggests that while bevacizumab is effective in achieving BCVA gains, there may be an increased treatment burden compared to aflibercept.

Of patients with ischaemic CRVO in our study, 20% developed neovascular complications within the study period. This is substantially lower when compared to the RAVE study which showed a 50% rate of neovascular complications [19]. Patients in the RAVE trial had a mean follow-up duration of 30 months with 65% completing the 36-month trial phase. The longer follow-up duration is likely to account for the difference in neovascular complication rates, particularly as the mean time to onset was 24 months, highlighting the need for continual monitoring in these high-risk patients. In addition, 70% of ischaemic CRVOs in our study were still receiving bevacizumab injections at the end of the study period. Another reason for this difference may be the differing definitions of ‘ischaemia’ creating a selection bias towards less severe forms of CRVO in our study.

Unsurprisingly, our multiple regression model showed that patients with a higher baseline BCVA achieved a lower mean visual acuity gain but had higher final visual acuities. Unlike previous models which showed poorer visual acuity outcomes associated with older age and greater treatment delay [20–23], our study was likely insufficiently powered to detect these associations.

This study had several limitations. It was a retrospective review with a small sample size, a lack of a control group, and used data collected in real-world practice. This may limit its generalisability. In addition, we selected patients who had a minimum of 6 months of treatment from the initial clinic visit, potentially selecting out patients with mild disease and leading to greater gains in BCVA. Our study has multiple strengths, including a mean follow-up duration of 18 months. Furthermore, patients in our study were treatment naïve and received no other treatment modalities (intravitreal steroid or macular laser) during the follow-up period which would have introduced confounding factors. Overall, this study is the first real-world evidence to support the use of off-label bevacizumab in a treat-and-extend regimen for patients with CMO secondary to CRVO.
